# "Whither Training in Psychiatry and Psychosomatic Medicine!" What need to be done ?

**Published:** 2004

**Authors:** C S Shamsundar

**Affiliations:** Bangalore


*Indian Journal of Psychiatry, 2004, 46(I)86*

### "Whither Training in Psychiatry and Psychosomatic Medicine!" What need to be done ?

Sir,

Inspite of repeated efforts by successive teams of Office Bearers of our IPS, due recognition by the IMC about the importance of Psychiatric and Psycho-social factors in both Under-graduate and Post-graduate Medical Education nothing much has been achieved.

It is not advisable any longer to keep up our efforts in this regard at the same pace as we have been doing till now. It is now imperative for us to take up this matter as an on-going policy issue in an aggressive manner. Towards this end, I have a few suggestions to make for the attention of the members of our profession as well as the current Office Bearers of our IPS.

1. Our (IPS) priority objective must be to get the Medical Council of India to officially acknowledge the paramount importance of 'Psychiatric' (Psycho-socio-biological) training not only in Under-graduate Medical education, but also in Post-graduate Training in all disciplines (including in such subjects as Anatomy, Physiology or Pathology) with the premise that, 'all medicine is Psycho-somatic medicine'. Once this objective is fulfilled, rest of the successive steps will become very easy. I will elaborate my points about this objective and its fulfillment in a few successive stages.

2. Apart from the well recognized 'psycho-somatic syndromes', almost all known conditions requiring medical intervention (under whatever discipline) have important psycho-social correlates either in etiology, exacerbation, complications, or effective managements or out-come. From this point of view, it can be said that 'all medicine is psycho-somatic medicine'. Every one of us know the truth of this fact; but, evidences to this effect will have to be generated and made available to: (i) professional leaders of all sister-medical disciplines, and (ii) office bearers of the Indian Medical Council.

3. The only way of achieving this objective (item 2) is by repeated clinical investigations or studies (in all clinical disciplines) about; (i) prevalence of psychiatric morbidity in patient population in each clinical discipline; (ii) psychosocial aspects of etiological factors; (iii) role of psycho-social factors and interventions for favourable prognosis, and (iv) publications of these investigations in National Journals of respective disciplines.

4. For achieving the above tasks, the responsibility of taking the initiative and continued active role has to be borne by mental health professionals, psychiatrists, psychologists, and psychiatric social workers, and psychiatric nurses. Of course, active participation of colleagues from the corroborating disciplines will have to be ensured including guideship and authorship.

5. One of the effective ways of achieving large number of studies or investigations as mentioned above is by providing good incentives for the professionals to take up such studies in earnest in large numbers: (i) Large pharmaceutical companies and other corporate bodies can be prompted (induced) to institute attractive prizes for well conducted studies each year (viz: Rs. 10,000 first prize, Rs. 5,000 second prize etc). (ii) separate sets of prizes to be instituted for each clinical discipline (viz: separately for medicine, surgery, obstetrics and gynecology, orthopaedics, paediatrics, dermatology, etc., etc). (iii) A body of experts (from non-involved discipline and Institute) can judge the quality of the studies for the purpose of prizes.

6. After some years, when convincingly large body of facts (about role of Psycho-social factors in etiology and management in all medical conditions) become available, an official representation should be made to the Medical Council of India about the required measures like: (i) due coverage of Psycho-social subjects in Undergraduate Medical Education, and (ii) the Post-graduate students (of any medical discipline) to have a three-months mandatory posting in a clinical psychiatric unit.

7. Of course, these above objectives will take some years to achieve. Therefore, it is very essential : (i) for the IPS to declare this issue as an on-going policy(till the objective is achieved); (ii) to make a start as immediately as possible; (iii) for the successive teams of Office-bearers to sustain the on-going activity with regular monitoring with equal commitment and energy, if not higher, and (iv) to attempt to actively involve the professional bodies (at national level) of sister disciplines like clinical psychology, psychiatric social work and Psychiatric nursing as well as the Armed Forces Medical Services.

**Dr. C.S. Shamsundar**, Bangalore

*Ed.* : We welcome response by the esteemed members.

